# Interaction between Hippocampal and Striatal Systems Predicts Subsequent Consolidation of Motor Sequence Memory

**DOI:** 10.1371/journal.pone.0059490

**Published:** 2013-03-22

**Authors:** Geneviève Albouy, Virginie Sterpenich, Gilles Vandewalle, Annabelle Darsaud, Steffen Gais, Géraldine Rauchs, Martin Desseilles, Mélanie Boly, Thanh Dang-Vu, Evelyne Balteau, Christian Degueldre, Christophe Phillips, André Luxen, Pierre Maquet

**Affiliations:** 1 Cyclotron Research Centre, University of Liège, Liège, Belgium; 2 University of Lyon, Lyon, France; Harvard Medical School, United States of America

## Abstract

The development of fast and reproducible motor behavior is a crucial human capacity. The aim of the present study was to address the relationship between the implementation of consistent behavior during initial training on a sequential motor task (the Finger Tapping Task) and subsequent sleep-dependent motor sequence memory consolidation, using functional magnetic resonance imaging (fMRI) and total sleep deprivation protocol. Our behavioral results indicated significant offline gains in performance speed after sleep whereas performance was only stabilized, but not enhanced, after sleep deprivation. At the cerebral level, we previously showed that responses in the caudate nucleus increase, in parallel to a decrease in its functional connectivity with frontal areas, as performance became more consistent. Here, the strength of the competitive interaction, assessed through functional connectivity analyses, between the caudate nucleus and hippocampo-frontal areas during initial training, predicted delayed gains in performance at retest in sleepers but not in sleep-deprived subjects. Moreover, during retest, responses increased in the hippocampus and medial prefrontal cortex in sleepers whereas in sleep-deprived subjects, responses increased in the putamen and cingulate cortex. Our results suggest that the strength of the competitive interplay between the striatum and the hippocampus, participating in the implementation of consistent motor behavior during initial training, conditions subsequent motor sequence memory consolidation. The latter process appears to be supported by a reorganisation of cerebral activity in hippocampo-neocortical networks after sleep.

## Introduction

The acquisition of reproducible motor sequence behavior represents a crucial human capability that is part of our everyday life activities (e.g., typing on a computer keyboard). Performance changes have been related to transitions between two types of processing modes: an initial and controlled mode followed eventually by a more automatic mode [1]–[3]. More particularly, consistency of performance, i.e. movement reproducibility, has been described to follow a specific time course during initial motor sequence learning and to reflect the implementation of a preferential, more effective and automatic performance mode [1], [2]. While cerebral correlates of motor sequence learning have been extensively studied and mainly involve cerebello-striato-cortical networks [2], [4]–[6], neural correlates of performance variability during motor sequence learning have only recently been explored. We previously demonstrated that, during initial motor sequence learning, responses in the caudate nucleus increased, whereas responses in the precuneus decreased, as performance became more consistent [2]. More particularly, the implementation of this preferential performance mode which eventually ensured the consistency of sequential motor output was related to functional interactions within striato-frontal and hippocampo-neocortical networks during early learning [2]. The potential impact of these early representations on the subsequent consolidation of motor sequence memory is an important issue that remains unexplored.

Memory consolidation represents the protracted process by which fresh, initially labile, memories are reorganized into stable memories [7]. At the behavioral level, motor skill consolidation is often characterized either by a reduction in the vulnerability of a recently acquired ability to the acquisition of a novel, interfering skill or by a spontaneous improvement in performance observed between practice sessions in the absence of further training [8], [9]. In motor sequence learning, substantial offline gains in performance have been reported several hours after training [10], [11]. In some cases, these performance gains are observed only if the interval contains a period of sleep [10], [12]–[14]. At the cerebral level, sleep-dependent motor sequence memory consolidation has been associated with changes in activity in cortical networks including prefrontal, motor and parietal areas [12], [14], as well as the striatum [15]. More specifically, using an oculomotor serial reaction time task [16], we showed that hippocampal and striatal responses during initial training predicted the overnight, possibly sleep-dependent, gain in performance observed 24 hours after training, but not the improvement of performance observed over the day, 30 minutes or 5 hours after training [5]. These early hippocampal responses may act as a tag for the neuronal populations that participate in offline memory processing during subsequent sleep. Interestingly, the competitive interaction observed during initial training between the striatum and the hippocampus turned to a cooperative interplay overnight, but not over the day, and may participate in the optimization of motor sequence behavior when the memory trace is consolidated [5].

The aim of the present study was to address the relationship between the implementation of reproducible motor behavior during initial training and subsequent sleep-dependent motor sequence memory consolidation. We hypothesized that the early representations underlying the achievement of consistent motor behavior influence subsequent sleep-dependent motor sequence memory consolidation. In addition, we took into account recent research suggesting that the sleep-dependent performance gains observed in motor sequence learning are influenced by a gradual buildup of fatigue over the course of massed practice [17], [18]. This can negatively affect performance during late training and lead to the overestimation of overnight performance changes. When fatigue is controlled for, the sleep enhancement effect is substantially reduced, suggesting that sleep does not enhance but only stabilizes motor performance. Importantly, this does not rule out a differential effect of sleep and sleep deprivation on performance and its neural correlates.

**Figure 1:**
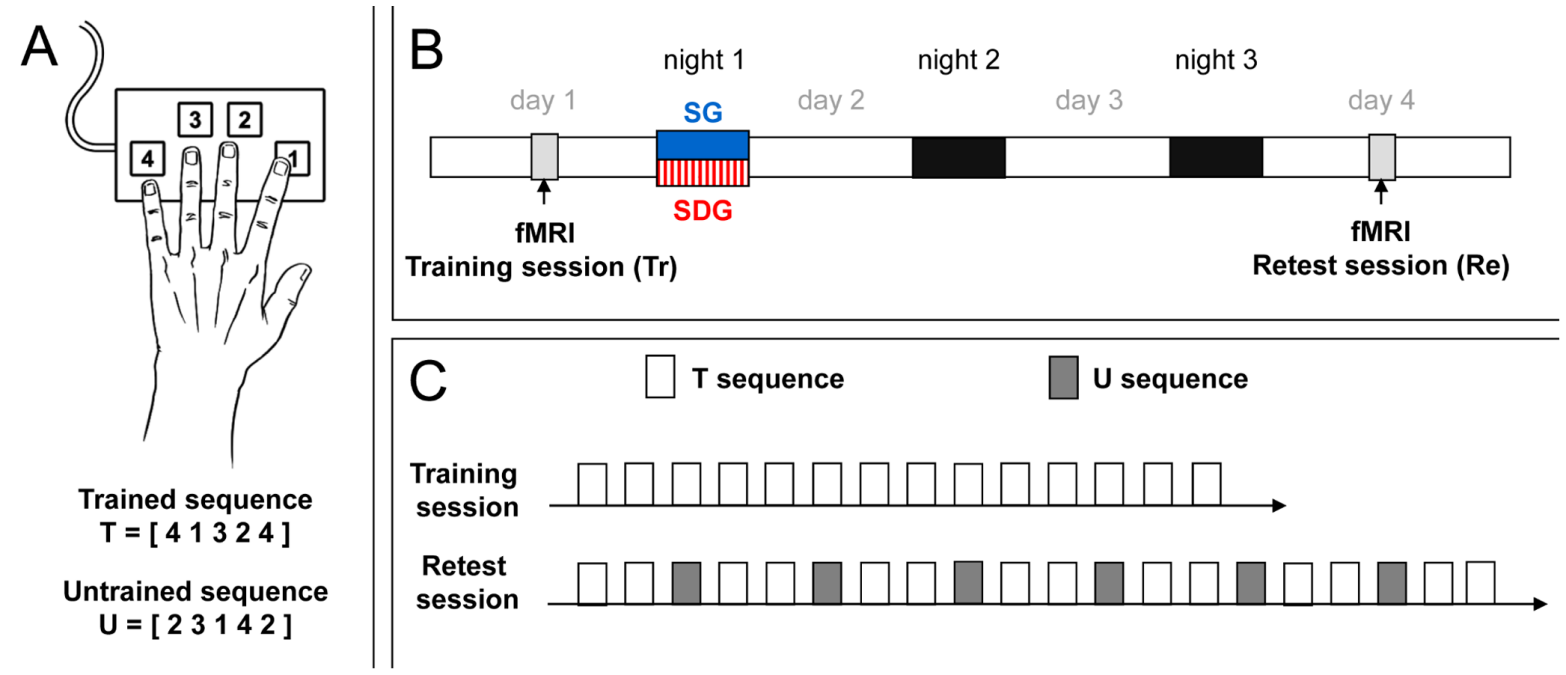
Experimental protocol. A- Finger Tapping Task, FTT. B- Experimental groups. Subjects were scanned during training and were divided in two groups according to the sleep condition on the first post-training night (SG: Sleep Group, SDG: Sleep Deprived Group). All the subjects were retested in the scanner three days later. C- Experimental design. Training and retest sessions consisted of 14 and 20 blocks respectively, each block consisting in 60 key presses. The untrained (U) sequence was proposed during retest, mixed with trained (T) sequence.

Using functional magnetic resonance imaging (fMRI), regional cerebral activity of participants was recorded during training on a sequential finger tapping task (Figure 1A). Subjects were divided in two groups after training depending on whether they slept (Sleep Group, SG) or were totally sleep-deprived (Sleep Deprived Group, SDG) during the first post-training night. In all cases, subjects slept as usual during the second and third post-training nights. Three days after training, during a second fMRI session, participants were retested on the motor task (Figure 1B). The impact of sleep and sleep deprivation on motor memory consolidation was indirectly revealed by changes in neural representation of motor memories during the retest session three days later.

## Materials and Methods

### Ethics Statement

All participants gave their written informed consent to take part in the study, which was approved by the Ethics Committee of the Faculty of Medicine of the University of Liège.

### Population

Thirty-four young (mean age: 23±3 years) right-handed [19] healthy volunteers were recruited by advertisement. They had no history of medical, neurological or psychiatric disease and none were on medication at the time of testing. No participants had ever played a musical instrument nor were trained as a typist. The quality of their sleep was normal as assessed by the Pittsburgh Sleep Quality Index questionnaire [20]. They followed a three-day constant sleep schedule (according to their own rhythm ±1 hour) before the first visit and kept the same schedule for three more days until their second visit. Compliance to the schedule was assessed using both sleep diaries and wrist actigraphy (Cambridge Neuroscience, Cambridge, UK).

### Task and General Experimental Design

Subjects were scanned during two separate sessions referred to as the training and retest sessions (Figure 1B) while they performed a finger tapping task (FTT) coded in Cogent2000 (http://www.vislab.ucl.ac.uk/cogent.php) and implemented in MATLAB (Mathworks Inc., Sherbom, MA). Results related to the training session were reported in a previous paper [2]. The FTT required the subjects to tap on a keyboard, with their (left) non-dominant hand, a 5-element finger sequence as rapidly and as accurately as possible (Figure 1A). The sequence to perform was explicitly known by the participants, constantly displayed on the screen and was one of two types: trained (T, 4 1 3 2 4) and untrained (U, 2 3 1 4 2). Training consisted of 14 successive practice blocks of the trained sequence separated by 15-second rest periods (Figure 1C). The task was coded to keep track of the number of key presses within a block (maximum 60 key presses). After 60 key presses, the “practice block” automatically turned into a “rest block” (fixation cross). Consequently, the duration of the practice blocks progressively decreased with learning as subjects became faster performing the 60 key presses (12 possible sequences). This protocol controlled for the number of movements executed per block to ensure that observed differences in cerebral responses were not contaminated by any change in motor output during practice.

After training, subjects were randomly assigned to one of two groups according to whether they were allowed to sleep (SG) or were totally sleep deprived (SDG) during the first post-training night (Figure 1B). Participants were informed of their assignment to the SG or the SDG only after the end of the training session. In the SG, subjects went home after the training session and slept regularly, as imposed by their constant sleep schedule, during the three post-training nights. In the SDG, subjects stayed awake in the laboratory during the first post-training night (from 11.00 p.m. to 7.00 a.m.). During this night, subjects remained under the constant supervision of experimenters and their physical activity was maintained as low as possible. Light was kept below 30 lux. Every hour, subjects were allowed to stand up and eat a small standardized snack. During the following day, subjects were instructed not to sleep and to continue their usual activities. They slept at home during the two remaining nights.

The retest session took place 72 h after training for subjects of both groups (SG and SDG) allowing two recovery nights for sleep deprived subjects (Figure 1B). Subjects were retested at different times of day ranging from 8 a.m. to 7 p.m. but training and retest sessions were conducted at the same time of day for each subject in order to account for possible circadian fluctuations in performance within subjects. The retest session consisted of 20 blocks, with 14 blocks of trained and 6 blocks of untrained sequences (Figure 1C). Two blocks of trained sequence were separated by one block of untrained sequence as follows (T T U T T U T T U T T U T T U T T U T T).

Motor skill performance was measured in terms of **speed** (block duration to perform 60 key presses), **error rates** (mean number of errors per block) and **variability.** Variability of performance was computed as the standard deviation of the residuals with respect to a single power-law fit that was calculated over the whole training session (i.e., over a maximum of 168 points representing the time to perform each correct sequence (12 possible correct sequences) over the 14 blocks of training) and the first two blocks of retest (i.e., over a maximum of 24 points in the first two blocks of retest). This method of variability analysis, adapted from Adi-Japha and collaborators [1], was used in our previous study [2] and implies that estimates of performance variability are orthogonal from performance speed estimates.

Supplemental fine-grained analyses on speed to perform each sequence within each block (mean response time between two key presses within a correct sequence [17]) were performed to assess possible fatigue effects. For this particular analysis, only the first 10 correct sequences (out of 12 possible correct sequences per block) were considered, because it represented, on average, the number of sequences that participants completed accurately (see accuracy paragraph in the results section).

### Behavioral Data Analyses

Speed, error rates and variability were computed for both training and retest sessions. A repeated-measures analysis of variance (ANOVA) on performance with block as a within-subjects factor and group (SG vs. SDG) as a between-subjects factor assessed the practice-related changes in performance during the training session. Another ANOVA on performance speed at the end of training was computed with block (average performance of the second last two blocks vs. average of the last two blocks of training) as a within-subjects factor and group (SG vs. SDG) as a between-subjects factor to test the saturation effect of learning at the end of training.

Between-session changes in performance, i.e. the offline gain in performance between the end of training and the beginning of retest, were tested with an ANOVA with block (average of performance on the last two blocks of training vs. average of the first two blocks of retest) as a within-subjects factor and group (SG vs. SDG) as a between-subjects factor.

Fatigue effects were explored by conducting repeated-measures ANOVAs on mean individual response times within a correct sequence [17] with block (14 practice blocks) and repetition of the trained sequence (10 sequences per block) as within-subjects factors and group (SG vs. SDG) as a between-subjects factor. Subsequent ANOVAs were conducted separately on each practice block in order to explore the effect of repetition of the sequence within block. Planned-comparisons were computed to test for the difference in response times between the first 5 sequences vs. the last 5 sequences on particular practice blocks (blocks 8, 13 and 14 of training and blocks 1 and 2 of retest, see Results section). To control for the possible influence of fatigue on the expression of between-session gains in performance, an ANOVA on blocks (average of response times on the first 5 sequences of the last two blocks of training vs. average of response times on the first 5 sequences of the first two blocks of retest) as a within-subjects factor and group (SG vs. SDG) as a between-subjects factor was computed.

### fMRI Data Acquisition and Analysis

Functional MRI-series were acquired using a head-only 3T scanner (Siemens, *Allegra*, Erlangen, Germany). Multislice T2*-weighted fMRI images were obtained with a gradient echo-planar sequence using axial slice orientation (TR = 2130 ms, TE = 40 ms, FA = 90°, 32 transverse slices, 3 mm slice thickness, 30% inter-slice gap, FoV = 220×220 mm^2^, matrix size = 64×64×32, voxel size = 3.4×3.4×3.0 mm^3^). Training and retest sessions consisted of 271±37 and 340±35 scans, respectively. A structural T1-weigthed 3D MP-RAGE sequence (TR = 1960 ms, TE = 4.43 ms, TI = 1100 ms, FA = 8°, 176 slices, FoV = 230×173 mm^2^, matrix size = 256×192×176, voxel size = 0.9×0.9×0.9 mm^3^) was also acquired in all subjects. Head movements were minimized using a vacuum cushion.

The three initial scans were discarded to allow for magnetic saturation effects. Functional volumes were pre-processed and analysed using SPM2 (http://www.fil.ion.ucl.ac.uk/spm/software/spm2/; Wellcome Department of Imaging Neuroscience, London, UK). Pre-processing included the realignment of functional time series, the co-registration of functional and anatomical data, a spatial normalization to an EPI template conforming to the Montreal Neurological Institute space, and a spatial smoothing (Gaussian kernel, 8 mm full-width at half-maximum, FWHM).

**Figure 2:**
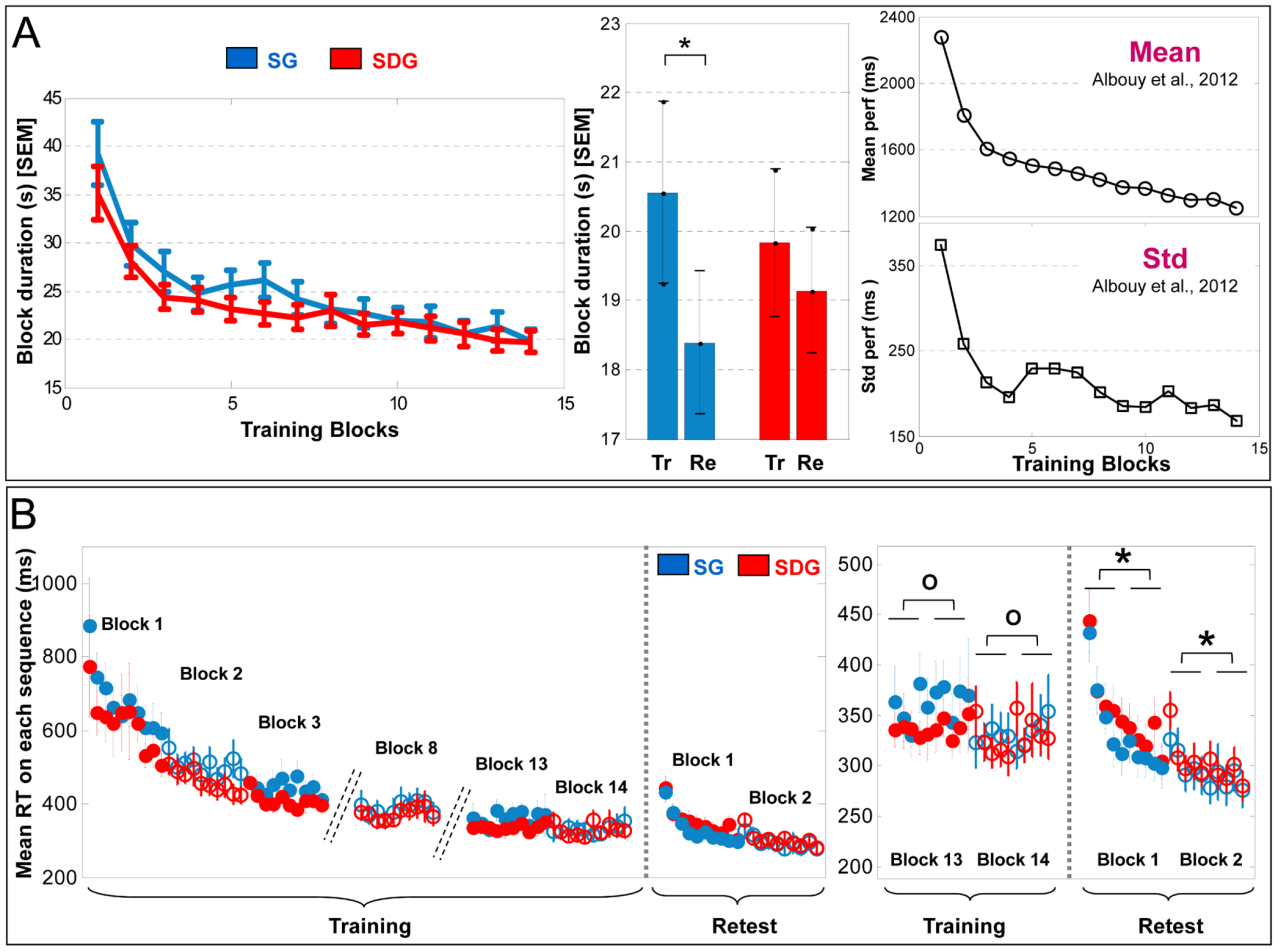
Behavioral results. Whiskers represent SEM. A- Left panel: Performance (mean block duration) improvement during training did not differ between the two groups. Middle panel: A significant ((*), p<0.05) offline gain in performance is observed in sleepers but not in sleep deprived subjects between the end of training (Tr) and the beginning of retest (Re). Right panel: Dynamics of mean time to perform a correct sequence (Mean, upper panel) and the standard deviation of difference between the data points (time to perform each correct sequence) and their power-law fit (Std, lower panel) computed over all subjects. Note that variability of performance follows a specific dynamics during training which does not parallel mean performance [2]. B- Left panel: Mean response time (RT) between two elements within a correct sequence for the first 10 correct sequences by block during both training and retest sessions. Note that the repetition effect is heterogeneous across blocks and that a significant fatigue effect manifests in block 8. Right panel: Between-session gains in performance are due to a rapid increase in RT during the retest session rather than to a slow-down in performance at the end of the training session ((*), p<0.05; (o), p>0.05).

The analysis of fMRI data, based on a mixed effects model, was conducted in two serial steps, accounting respectively for fixed and random effects. For each subject, changes in brain regional responses were estimated by a model including the responses to the trained sequence and their linear modulations by performance speed (mean time to perform a correct sequence by block, Figure 2A, right panel, Mean) and variability (standard deviation of the residuals with respect to a single power-law fit, per block, Figure 2A, right panel, Std). Variability was orthogonalized with respect to speed, to account for potential colinearity. Any significant brain region revealed by parametric modulation analyses by performance variability will present a dynamical BOLD response that is linearly (1^st^ order polynomial expansion) related to the (non-linear) pattern of performance variability changes (see Figure 2A, right panel, Std, to appreciate this non-linear dynamics). These regressors consisted of box cars convolved with the canonical hemodynamic response function. Movement parameters derived from realignment of the functional volumes were also included as covariates of no interest. High-pass filtering was implemented in the design matrix using a cut-off period of 128 seconds to remove slow drifts from the time series. Serial correlations in fMRI signal were estimated using an autoregressive (order 1) plus white noise model and a restricted maximum likelihood (ReML) algorithm.

For the training session, contrasts tested the main effect of practice of the trained sequence and its linear modulation by performance variability. Modulation by performance variability identified regions where response amplitude increased as motor behavior became more consistent (i.e., less variable) across training.

For the retest session, a linear contrast tested the main effect of practice of the trained sequence. Finally, a linear contrast tested the main effect of session (retest vs. training) on the practice of the trained sequence. These linear contrasts generated statistical parametric maps [SPM(T)]. The resulting contrasts images were then further spatially smoothed (Gaussian kernel 6 mm FWHM) and entered in a second-level analysis, corresponding to a random effects model, accounting for inter-subject variance.

**Figure 3:**
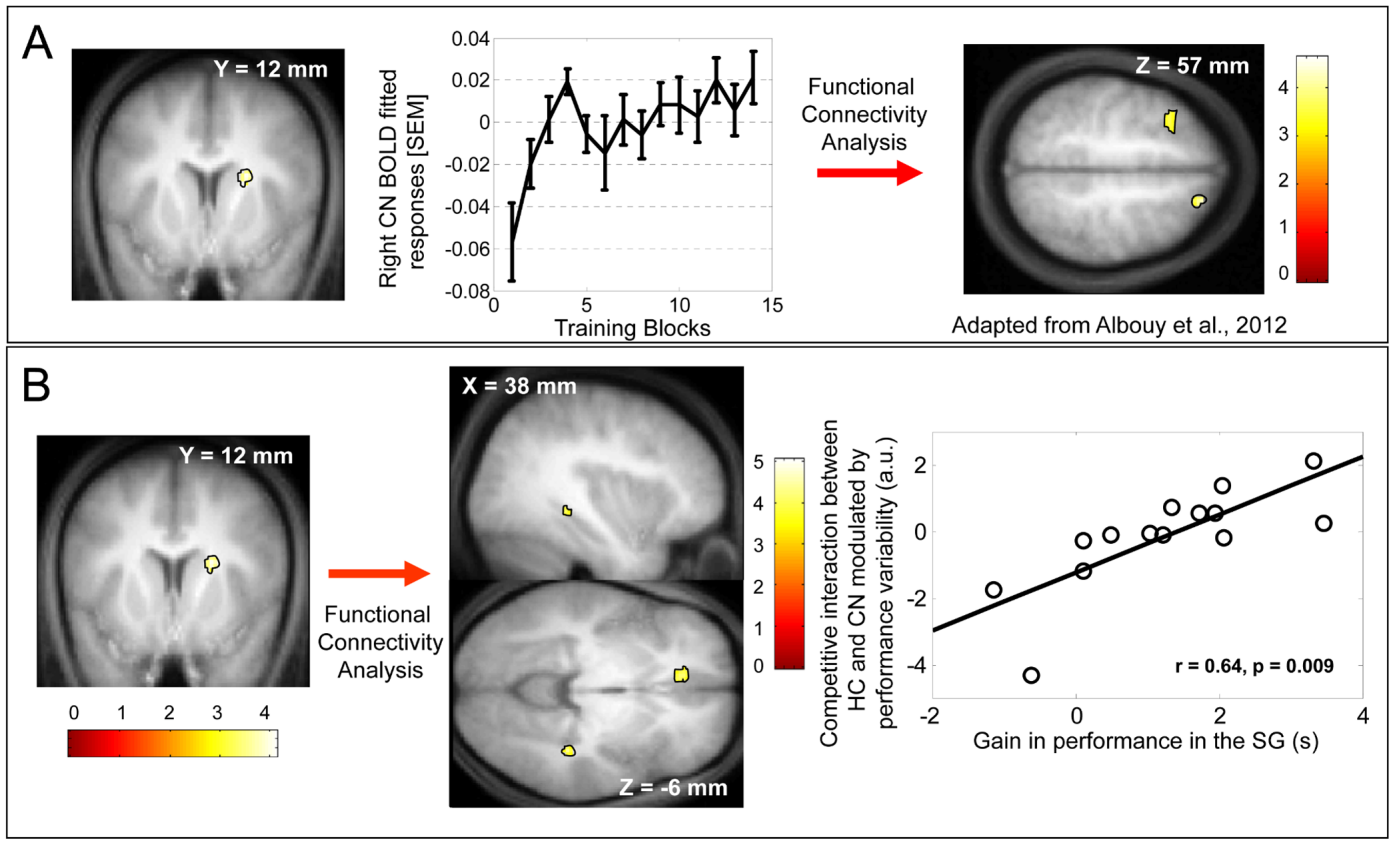
Functional imaging results for the training session. Functional results are displayed at p_uncorrected_<0.001 over the mean structural image of all subjects. In the insets, whiskers represent SEM. CN: Caudate Nucleus, HC: Hippocampus. A- Linear modulation of brain responses by performance consistency. Caudate nucleus responses increased during training in parallel to performance reproducibility. The dynamics of caudate activity follows a similar non-linear pattern as performance consistency during training. The functional connectivity between the caudate nucleus and frontal areas is proportional to performance variability during training [2]. B- Regression analysis between cerebral areas functionally connected with the caudate nucleus, in proportion to performance variability and gain in performance in the SG. Left panel: The strength of the functional connectivity (competitive interaction) between the caudate nucleus and hippocampo-cortical areas is correlated with the subsequent gains in performance on the learned sequence in the SG. Right panel: Regression plot of the strength of the functional connectivity (competitive interaction) between the caudate nucleus and the hippocampus related to performance variability against the gains in performance in the SG (block duration (s)) on the learned sequence. Each data point represents a single subject of the SG.

Regarding second level analyses, for the training session, one-sample t tests were run on the data of all the subjects as this session was identical for both groups. A first analysis characterized the main effect of practice of the trained sequence. A second analysis characterized the temporal dynamics of brain responses during training, based on their linear modulation by performance variability. Results related to this particular analysis are reported in a previous paper [2]. The fitted BOLD responses modulated by performance were estimated to illustrate the block by block temporal dynamics of cerebral responses in areas showing modulation of activity by performance variability (Figure 3A, caudate nucleus, adapted from [2]).

Psychophysiological interaction (PPI) analyses were computed to test the functional connectivity of the right caudate nucleus with the rest of the brain, in proportion to performance variability changes during training. New linear models were generated at the individual level, using three regressors. One regressor represented the practice of the learned sequence modulated by performance variability. The second regressor was the activity in the reference area. The third regressor represented the interaction of interest between the first (psychological) and the second (physiological) regressors. To build these regressors, the underlying neuronal activity was first estimated by a parametric empirical Bayes formulation, combined with the psychological factor and subsequently convolved with the hemodynamic response function [21]. The design matrix also included movement parameters. A significant PPI indicated a change in the regression coefficients (i.e. a change in the strength of the functional interaction) between any reported brain area and the reference region (caudate nucleus), related to performance variability changes during training. These results are reported in our previous paper [2].

As the neural correlates of performance variability during initial motor sequence acquisition had never been characterized before, a separate paper has been published on this particular topic [2]. The results reported in the present study are original findings linking the implementation of reproducible performance and subsequent motor sequence memory consolidation. We then performed an analysis assessing the relationship between the functional connectivity of the caudate nucleus, in proportion to performance variability during training, and the subsequent gain in performance on the trained sequence (controlled or not for fatigue effects) observed between training and retest sessions. We then regressed the contrast images of the individual functional connectivity of the caudate nucleus modulated by variability of performance against the offline gain in performance on the trained sequence (controlled or not for fatigue effects), separately for each group (SG and SDG). A final two sample t test compared these regressions between the two groups (SG vs. SDG).

For the retest session, one sample t tests were run separately for each group. A first analysis characterized the main effect of practice of the trained sequence in both groups (SG and SDG). A conjunction analysis based on a conjunction null hypothesis characterized brain areas jointly activated in both groups (SG and SDG).

A final analysis compared the main effect of practice of the trained sequence between sessions (retest vs. training) for each group. This analysis characterized the changes in brain responses to the trained sequence between training and retest sessions. Exclusive masks (EM) were used to isolate the effects specific to each group.

The resulting set of voxel values for each contrast constituted a map of the t statistic [SPM(T)], thresholded at p<0.001 (uncorrected for multiple comparisons). Statistical inferences were performed at a threshold of p<0.05 after correction for multiple comparisons over either the entire brain volume or over small spherical volumes (10 mm radius), located in structures of interest, reported by published work on motor learning.

### Coordinates of Areas of Interest Used for Spherical Small Volume Corrections

Coordinates used for spherical small volume corrections were located in areas already reported for their involvement in motor sequence learning and consolidation (striatum, hippocampus, cerebellum, as well as motor, cingulate, frontal, temporal and parietal cortices). The papers from which these coordinates of interest were extracted are listed below with some indication on the task and the type of design used:

[5], Task: Serial oculomotor reaction time task, Design: Training session followed by retest sessions occurring either 30 min, 5 h or 24 h after initial training; [22], Task: Audio-paced finger tapping task, Design: Training session with different movement complexity and frequency; [23], Task: Timed motor sequence learning task, Design: Training session with learned, isochronous and perceptual sequences; [14], Task: Finger Tapping Task, Design: Training session followed by regular sleep or sleep deprivation and a 48 h retest session; [24], Task: Timed motor sequence learning task, Design: Training session on learned and isochronous sequences (day 1), retest session after 5 days of practice (day 5) and recall after four weeks; [25], Correction for the MPFC activation, described for sleep-dependent consolidation of declarative memories, Task: Emotional memory, Design: Encoding of neutral or emotional images followed by a regular night of sleep or total sleep deprivation and a 72 h memory retest; [26], Task: Serial reaction time task, Design: Implicit or explicit sequence learning followed by generation tasks testing the awareness of the sequence.

The coordinates selected from these studies are listed below:

*Striatal locations* : right ventral putamen 26 4–24 mm; right caudate nucleus 18 8 20 mm [5]; left posterior putamen −24.6±3.6 −0.6±5.9 3.4±9.0 mm [22]; *Cerebellar locations:* left cerebellar hemisphere −22 −64 −26 mm [23], −18 −44 −18 mm [14], right cerebellar hemisphere 22 −38 −36 mm [5]; *Hippocampal locations:* left anterior hippocampus −16 −14 −28 mm, −34 −10 −20 mm [5]; right posterior hippocampus 42 −34 −12 mm, 26 −34 −4 mm [5]; *Cingulate cortex:* posterior cingulate −4 −48 34 mm [24]; anterior cingulate cortex 2 48 12 mm [23]; *Frontal cortex locations:* medial prefrontal cortex −6 60 2 mm, 10 50 14 mm [25], −4 36 −8 mm [26]; left superior frontal gyrus −12 36 56 mm [24], −14 26 54 mm, −32 14 50 mm [23]; right superior frontal gyrus 44 15 46 mm [5], 32 54 22 mm, 18 40 46 mm, 14 26 54 mm [23]; right medial frontal cortex 48 28 46 mm [5]; *Temporal cortex locations:* right medial temporal gyrus 62 −8 −28 mm [23]; inferior temporal gyrus ±42 −8 −38 mm [24], 54 −22 −16 mm [23]; *Motor cortex locations*: sensorimotor cortex ±36.2±3.0 −22.3±4.6 57.0±6.1 mm [22]; supplementary motor area +/−2 −2 70 mm; primary motor cortex −14 −20 74 mm, 10 −22 58 mm; left premotor cortex −12 0 74 mm [23]; *Parietal cortex locations:* right parietal cortex 50 −54 42 mm [23]; right intraparietal sulcus 30 −54 70 mm [5].

## Results

### Population

Four subjects were discarded from the analyses because of large movements during the acquisition (1 in each group) or because they practiced a wrong sequence in the scanner (1 in each group). Eventually, 30 subjects were included in the analyses, 15 subjects in each group (SG: mean age = 23±2.2 years, 7 females; SDG: mean age = 23.6±2.8 years, 8 females).

### Subjective Assessment of Sleep Duration and Quality

The groups did not differ in mean sleep duration (SG, 8 h 12 min ±1 h 07 min; SDG, 7 h 42 min ±42 min; unpaired *t* test, *t*_(28) = _1.37, p = 0.18) or in the median Pittsburgh Sleep Quality Index (PSQI) score (SG, 3; SDG, 4; unpaired *t* test, *t*_(28) = _0.38, p = 0.70) over the month preceding the recordings. Sleep duration and quality, subjectively assessed using the St. Mary’s Hospital sleep questionnaire [from very poor (1) to good (5)], did not differ between groups during the night preceding the training session (Duration: SG, 7 h 30 min ±57 min; SDG, 7 h 42 min ±56 min; unpaired *t* test *t*_(28)_ =  −0.39, p = 0.69; Quality: SG, 4; SDG, 4; unpaired *t* test *t*_(28)_ = 0.68, p = 0.50) or during the night preceding the retest session (Duration: SG, 7 h 45 min ±1 h 19 min; SDG, 8 h 09 min ±1 h 18 min; unpaired *t* test *t*_(28)_ =  −0.89, p = 0.37; Quality: SG, 3; SDG, 4; unpaired *t* test *t*_(28)_ =  −1.70, p = 0.10).

### Actigraphic Data

Actigraphic data were collected by wrist actigraphy (Cambridge Neuroscience, Cambridge, UK) during 6 days (three days before and after the training session) where subjects followed a constant sleep schedule. A repeated-measures ANOVA on actigraphic activity with nights (6 nights) as a within-subjects factor and group (SG vs. SDG) as a between-subjects factor showed significant main effects of group (F(1,28) = 43.88, p<0.001) and night (F(5,140) = 76.79, p<0.001) as well as a group by night interaction (F(5,140) = 67.41, p<0.001). The activity during the first three nights did not differ between groups (all p_s_ >0.3). Sleep duration, estimated jointly with sleep diaries and actigraphic recordings, did not differ during the night preceding the training session (SG, 7 h 56 min ±0 h 54 min, SDG 8 h 00 min ±0 h 48 min, unpaired t test t_(28)_ = 0.00, p = 1.00, see also “*Subjective assessment of sleep duration and quality*” paragraph). As expected, the activity was larger in the SDG than in the SG during the deprivation night (SG = 38.87±33.59 activity units, SDG = 274.52±97.04 units, F(1,28) = 78.98, p<0.001). Actigraphic and sleep diaries data indicated that subjects of the SG slept, at home, an average 8 h 56 min ±1 h 16 min during the first post-training night (sleep duration ranging from 7 to 10 hours). During the first recovery night, activity in the SDG tended to be lower than in the SG, suggesting a rebound of sleep after sleep deprivation (SG = 34.52±13.77 units, SDG = 25.04±14.33 units, F(1,28) = 3.40, p = 0.07). Sleep duration also tended to be larger in the SDG as compared to the SG (SG, 8 h 44 min ±1 h 18 min; SDG, 9 h 46±1 h 34 min, unpaired t test t_(28)_ =  −1.96, p = 0.06). These effects were not present on the second recovery night, which preceded the retest session (Activity, SG = 37.47±21.38 units, SDG = 31.00±17.52 units, F(1,28) = 0.82, p = 0.37; Sleep duration, SG, 8 h 22 min ±1 h 19 min; SDG, 8 h 16±1 h 28 min, unpaired t test t_(28)_ = 0.19, p = 0.84, see also “*Subjective assessment of sleep duration and quality*” paragraph), suggesting that two nights were sufficient to recover from the effects of the sleep deprivation.

Actigraphic data during daytime (5 days) showed no significant main effects of group (F(1,28) = 0.41, p = 0.83) and day (F(4,112) = 1.88, p = 0.11) as well as no group by day interaction (F(4,112) = 0.06, p = 0.99). The activity during the day following the sleep deprivation did not differ between groups (SG = 347.93±99.88 units, SDG = 350.23±103.37 units, F(1,28) = 0.003, p = 0.95), suggesting that sleep deprived subjects maintained the same level of activity than sleepers the day after the sleep deprivation.

### Behavioral Results

Training consisted of 14 blocks of practice of the trained sequence. The retest session took place 72 hours later and consisted of 20 blocks, with 14 blocks of trained interleaved with 6 blocks of untrained sequences (see Methods and Figure 1C). In the following paragraphs, we focus on performance speed, accuracy, variability as well as the influence of fatigue.

#### Performance speed during training session

For the training session, an ANOVA conducted on performance speed with blocks of trained sequence (14 practice blocks) as a within-subjects factor and group (SG vs. SDG) as a between-subjects factor showed a main effect of block (F(13,364) = 52.04, p<0.0001) whereby block duration decreased with practice in both groups. In contrast, there were no significant group effect (F(1,28) = 0.57, p = 0.45) and no significant repetition by group interaction (F(13,364) = 1.14, p = 0.32), indicating that subjects of both groups similarly improved on the trained sequence during training (Figure 2A, left panel).

#### Between-session gains in performance speed

Between-session effects were computed comparing the average of the last two blocks of the training session against the first two blocks of the retest session in order to assess offline improvement. The ANOVA revealed a significant main effect of session (F(1,28) = 5.01, p = 0.03), but no significant group effect (F(1,28) = 0.0001, p = 0.9) or group by session interaction (F(1,28) = 1.35, p = 0.25). However, planned comparison showed a significant effect of session in the sleep group (F(1,14) = 6.72, p = 0.02), indicating that subjects who slept after training presented significant offline improvement. The delayed gain observed in sleepers is not likely to be due to a continuation of the initial learning process as asymptotic performance was reached at the end of training: The ANOVA testing the saturation effect did not reveal significant improvement over the last four blocks of training (F(1,14) = 1.38, p = 0.25). In contrast, no significant effect of session was observed in the sleep deprived group (F(1,14) = 0.50, p = 0.48), indicating that subjects who were sleep deprived during the first post-training night did not present any significant offline improvement (Figure 2A, middle panel).

#### Accuracy during training session

For the training session, an ANOVA conducted on the number of errors per block (i.e. error rate) with repetition of the trained sequence (14 blocks of trained sequence) and group (SG vs. SDG) as factors did not show significant main effects of repetition (F(13,364) = 1.04, p = 0.40), as the mean number of errors remained stable and low (1.05±1.32 errors per blocks) throughout training. There were no significant group effect (F(1,28)<0.001, p = 0.98) and no significant repetition by group interaction (F(13,364) = 0.62, p = 0.83), indicating that subjects of both groups had similar error rates during training.

#### Between-session gains in performance accuracy

Between session effects were computed comparing the average of the last two blocks of the training session against the first two blocks of the retest session in order to assess offline improvement. The ANOVA revealed no significant effect of session (F(1,28) = 0.16, p = 0.68), no significant group effect (F(1,28) = 0.62, p = 0.43) and no group by session interaction (F(1,28) = 0.75, p = 0.39). Furthermore, planned comparison did not show any significant effect of session in both the sleep and sleep deprived groups (SG: F(1,14) = 0.40, p = 0.53; SDG: F(1,14) = 0.46, p = 0.50).

#### Performance variability during training session

For the training session, a repeated-measures ANOVA conducted on the variability of the power law fit residuals with block as a within-subjects factor and group (SG vs. SDG) as a between-subjects factor showed a main effect of block (F(13,364) = 5.68, p<0.0001), indicating that performance variability significantly changed across training blocks. In contrast, there was no significant group effect (F(1,28) = 1.45, p = 0.23) or significant repetition by group interaction (F(13,364) = 0.96, p = 0.48), indicating that subjects of both groups had similar changes in performance variability during training. As shown in Figure 2A (right panel, Std) performance became progressively more consistent for all subjects across blocks (i.e., standard deviation decreased), except during blocks 5 to 7 during which behavior became temporarily more variable. This precise time course, detailed in our previous paper [2], is strikingly similar to the change in performance consistency reported for the finger opposition task by Adi-Japha and colleagues [1] and occurred independently of concurrent changes in performance speed (Figure 2A, right panel, Mean).

#### Between-session changes in performance variability

Between-session effects were computed comparing averaged performance variability of the last two blocks of training against the first two retest blocks. The ANOVA revealed no significant main effect of session (F(1,28) = 1.47, p = 0.23), no significant group effect (F(1,28) = 0.94, p = 0.33) and no group by session interaction (F(1,28) = 0.04, p = 0.82), indicating that movement reproducibility was maintained from training to testing in both groups.

#### Fatigue effects during training session

Fine-grained analyses of performance speed were conducted for each sequence within each block in order to determine the influence of fatigue on between-session gains in performance speed.

An ANOVA conducted on performance speed (i.e., mean response time between two elements within a correct sequence [17]) with block (14 practice blocks) and repetition of the trained sequence (10 sequences per block) as within-subjects factors and group (SG vs. SDG) as a between-subjects factor showed significant effects of block (F(13,338) = 54.41, p<0.001) and sequence (F(9,234) = 1.88, p = 0.05) as well as a significant block by sequence interaction (F(117,3042) = 2.74, p<0.001). The effect of group was not significant (F(1,26) = 0.91, p = 0.34). The repetition effect was heterogeneous across blocks (Figure 2B, left panel). Indeed, subsequent ANOVAs conducted on each practice block showed that performance speed improved across sequences within each block during the first four training blocks (all F(9,252)>2.27, all p<0.02). In contrast, no repetition effects (all F(9,252) <1.71, all p>0.05) were observed in the other training blocks of the session (blocks 5–7 and 9–14), except on block 8 during which performance speed worsened across sequence repetition, suggesting an effect of fatigue in this block (F(9,252) = 1.9, p = 0.05, Figure 2B, left panel, Block 8). Planned comparison within this block indicated that this fatigue effect occurred in the second half of the block whereby a significant deterioration of performance (F(1,27) = 5.46, p = 0.02) was observed between the first 5 vs. the last 5 sequences.

In conclusion, our data do not show a consistent worsening of performance due to repetition of 10 sequences within the last training blocks. These results could suggest that the repetitive practice of the motor task did not induce any significant fatigue effect at the end of training. On the other hand, a more probable explanation is that the potentially detrimental effects of fatigue on performance during late training result in stabilization of performance speed (as opposed to the improvement in speed observed within the early blocks that are less affected by fatigue). This explanation could not be distinguished from a practice-dependent plateau-effect.

#### Between-session gains in performance controlled for possible fatigue effects

Despite the absence of clear effects of fatigue on performance during training, between-session gains in performance were re-computed with the sequences that are not affected by possible fatigue effects (5 first sequences within each block).

The ANOVA revealed neither a significant main effect of session (F(1,28) = 2.03, p = 0.16), nor a significant group effect (F(1,28) = 0.0009, p = 0.98) nor a significant group by session interaction (F(1,28) = 1.52, p = 0.22). These results confirm the recent behavioral studies [17], [18] reporting that delayed gains in performance are less robust when controlled for fatigue than otherwise. Nevertheless, planned comparisons still showed a significant effect of session in the sleep group (F(1,14) = 4.50, p = 0.05) but no significant effect of session in the sleep deprived group (F(1,14) = 0.014, p = 0.90). These results suggest that the significant between-session improvement in performance observed in sleepers is not entirely explained by a passive dissipation of fatigue.

Further analyses showed that the overall overnight gains in performance were more due to a significant improvement on the last 5 sequences during the first two retest blocks than to a worsening on the last 5 sequences of the last two training blocks (Figure 2B, right panel). Indeed, for the last two blocks of training, performance on the last 5 sequences did not differ from performance on the first 5 sequences (SG, F(1,14) = 0.82, p = 0.37 and SDG, F(1,14) = 1.84, p = 0.18, Figure 2B, right panel). In contrast, on the first two retest blocks, performance on the last 5 sequences was significantly better that on the first 5 sequences (SG, F(1,14) = 19.24, p<0.001 and SDG, F(1,14) = 24.65, p<0.001, Figure 2B, right panel), indicating a strong improvement in performance within the first two retest blocks.

To conclude, the data suggest that in this case, no significant worsening in performance, usually considered as the expression of fatigue [17], [18], was observed at the end of the training session. However, our results cannot dismiss the influence of fatigue as it could manifest itself at the end of training by a stabilization rather than an impairment of within-block performance. Importantly, gains in performance remained significant in the SG after controlling for fatigue, indicating that this specific effect was due to an active mnemonic process rather than to a passive dissipation of fatigue [17], [18]. Interestingly, these fine grained analyses also showed that the overnight gain in performance seems to be due to a strong increase in performance within the first blocks of the retest session.

#### Time of testing

Training and retest sessions were conducted from 8 a.m. to 7 p.m. across participants and were performed at the same time of day for each subject in order to account for possible circadian fluctuations in performance within subjects. From the 30 subjects included in the analyses, 15 were tested during the morning (from 8 to 12 a.m, 8 in the SG, 7 in the SDG) and 15 during the afternoon (from 1 to 7 p.m., 7 in the SG, 8 in the SDG). Unpaired t-tests indicated that the average time of testing did not differ between groups (SG: 13 h 13 min ±0 h 51 min and SDG: 13 h 55 min ±0 h 58 min, unpaired t-test t_(28)_ =  −0.53, p = 0.59).

Nevertheless, time of testing was entered as a covariate in an ANCOVA examining the effects of session (average of the last two blocks of training vs. first two blocks of retest session, not controlled for fatigue) and group (SG vs. SDG). No significant covariate (F(1,27) = 0.46, p = 0.49) or covariate by session effects (F(1,27) = 1.60. p = 0.21) were observed. The session by group interaction remained non-significant (F(1,27) = 1.60, p = 0.21) but within group analyses still indicated significant gains in performance in the SG (p = 0.019) that were not observed in the SDG (p = 0.49). These results indicate that the changes in performance speed that were observed between training and retest sessions in both groups were not significantly modulated by the time of testing.

### Brain Imaging Data

Practice of the learned sequence during training and retest sessions recruited a large cerebello-cortical network as reported in Table 1.

**Table 1:** Functional results for the practice of the trained sequence during training and retest sessions.

Area	x mm	y mm	z mm	Z	p
**Practice of the trained sequence during training**					
Right Motor Cortex	36	−18	62	Inf	**0.000**
Left Motor Cortex	−50	−24	48	6.75	**0.000**
	−32	−6	68	6.55	**0.000**
	−60	6	28	9.53	**0.000**
Left Cerebellar Lobule V/VI	−18	−50	−26	Inf	**0.000**
	−4	−58	−12	7.69	**0.000**
Right Cerebellar Lobule V/VI	24	−60	−24	7.43	**0.000**
Right Globus Pallidus	16	−6	−8	5.20	**0.005**
Left Globus Pallidus	−16	−8	−4	4.87	**0.021**
Left Intraparietal Sulcus	−26	−52	68	5.44	**0.002**
Right Intraparietal Sulcus	32	−50	72	5.82	**0.000**
Right Cingulate Motor Area	2	2	56	6.20	**0.000**
**Conjunction of SG and SDG for the practice of the trained sequence during retest**					
Left Cerebellar Lobule V	−16	−50	−22	7.18	**0.000**
Left Cerebellar Lobule V/VI	−4	−50	−12	6.92	**0.000**
Left Cerebellar Lobule VI	−20	−62	−22	5.99	**0.000**
Right Motor Cortex	36	−18	70	6.24	**0.000**
	50	−22	60	6.24	**0.000**
	40	−32	70	6.05	**0.000**

Only significant brain responses after correction over the entire volume are reported.

#### Modulation of cerebral activity by performance variability during training

During training, performance variability was considered as a potentially important modulator of brain responses because it quantifies the ability to maintain a reproducible performance level within a block [2]. Modulation analyses show that the amplitude of the cerebral responses increased in the right caudate nucleus as performance became more consistent, i.e., as variability of the residuals with respect to the power law fit decreased (Table 2–1, results reported in [2]). The time course of responses in this area followed a non-linear pattern that closely paralleled the evolution of performance variability and was characterized by a decrease in activity at mid-training (Figure 3A left panel, adapted from [2]). Furthermore, functional connectivity analyses revealed that the activity in the right caudate nucleus was coupled with a set of frontal areas, in proportion to performance variability. This result implies that the striato-frontal interaction was strong when performance was variable, diminished in proportion to the decrease in performance variability and was transiently strengthened at mid-training when performance was more variable (Figure 3A right panel, Table 2–2, results reported in [2]).

**Table 2:** Functional results for the training session.

Area	x mm	y mm	z mm	Z	p_svc_
**1- Cerebral areas where responses increase in proportion to decrease in variability ** [**2**]					
Right Caudate Nucleus	22	12	18	3.64	**0.004**
Right Motor Cortex	10	−24	56	3.98	**0.003**
**2- Functional connectivity of the right caudate nucleus modulated by performance variability ** [**2**]					
Right Superior Frontal Gyrus	22	38	54	4.05	**0.003**
	18	34	58	3.25	**0.030**
Left Superior Frontal Gyrus	−20	26	62	3.18	**0.036**
	−34	18	58	3.39	**0.021**
**3- Regression between functional connectivity of caudate nucleus modulated by performance variability and overnight gain in performance**					
**SG**					
Right Superior Frontal Gyrus	22	26	60	3.80	**0.006**
Left Medial Prefrontal Cortex	−10	36	−2	4.07	**0.003**
Right Anterior Cingulate Cortex	4	50	6	3.17	**0.038**
	4	40	8	3.14	**0.041**
Right Posterior Hippocampus	40	−38	−6	3.79	**0.006**
Right Middle Frontopolar Gyrus	24	58	14	3.48	**0.037**
Right Inferior Temporal Gyrus	54	−14	−22	3.45	**0.018**
**SDG**					
No Significant Responses					
**SG – SDG**					
Left Medial Prefrontal Cortex	−10	36	−2	3.74	**0.007**
Left Motor Cortex	−26	−12	64	3.41	**0.020**
Left Premotor Cortex	−8	−4	74	3.41	**0.020**
Right Posterior Hippocampus	32	−36	−4	3.18	**0.037**
**SDG – SG**					
No Significant Responses					
**4- Regression between functional connectivity of caudate nucleus modulated by performance variability and overnight gain in performance controlled for possible fatigue effects**					
**SG**					
Right Superior Frontal Gyrus	22	26	60	4.25	**0.001**
	20	32	58	3.92	**0.004**
Right Medial Frontal Gyrus	52	18	46	3.95	**0.004**
Left Medial Prefrontal Cortex	−8	36	−2	4.18	**0.002**
Right Medial Prefrontal Cortex	6	42	12	3.55	**0.013**
Right Inferior Temporal Gyrus	54	−14	−22	3.84	**0.005**
Right Cerebellar Lobule V	30	−34	−36	3.54	**0.014**
Left Cerebellar Lobule IV	−18	−34	−28	3.28	**0.029**
	−12	−42	−12	3.36	**0.023**
Right Middle Frontopolar Gyrus	26	58	14	3.64	**0.010**
Right Posterior Hippocampus	40	−38	−8	3.54	**0.014**
Left Anterior Hippocampus	−22	−16	−32	3.15	**0.040**
Left Putamen	−24	−2	−2	3.24	**0.033**
	−26	2	−10	3.14	**0.042**
Left Motor Cortex	−34	−26	70	3.17	**0.039**
**SDG**					
No Significant Responses					
**SG – SDG**					
Left Anterior Hippocampus	−22	−18	−32	3.55	**0.013**
Right Parietal Cortex	58	−50	38	3.48	**0.017**
Right Superior Frontal Gyrus	50	18	44	3.35	**0.024**
Left Primary Motor Cortex	−6	−20	70	3.48	**0.017**
Left Supplementary Motor Area	−8	−4	74	3.47	**0.017**
Right Supplementary Motor Area	16	−6	76	3.26	**0.031**
Left Medial Prefrontal Cortex	−10	36	−2	3.46	**0.018**
Right Medial Prefrontal Cortex	14	60	6	3.24	**0.033**
Right Intraparietal Sulcus	26	−62	66	3.42	**0.020**
Right Medial Frontal Gyrus	48	20	44	3.23	**0.033**
Right Posterior Hippocampus	32	−36	−4	3.15	**0.040**
**SDG – SG**					
No Significant Responses					

Significant brain responses after correction over small volume of interest (svc) are reported here. SG: Sleep Group; SDG: Sleep Deprived Group. Results presented in points 1- and 2- of this table have already been reported in [2].

Regarding the specific caudate recruitment and its functional interactions with the rest of the brain, we assessed whether its functional connectivity modulated by performance variability observed during initial training could be correlated with the subsequent gains in performance emerging after sleep but not after sleep deprivation. This regression analysis showed that the strength of the negative functional connectivity (competitive interaction) between the caudate nucleus and numerous cortical areas was linearly related to the delayed gain in performance speed in sleepers, and more so in sleepers than in sleep deprived subjects in whom no such regression was observed. This cerebral network consisted of a set of cortical areas including the superior frontal cortex, the medial prefrontal cortex, the middle frontopolar cortex, the anterior cingulate cortex, the inferior temporal gyrus and the hippocampus (Table 2–3). Figure 3B (left panel) shows the connectivity maps of the caudate nucleus, in proportion to the implementation of reproducible motor behavior, and in relation to subsequent gains in performance observed in the SG as compared to the SDG. The right panel of the figure shows how the strength of the functional connectivity (competitive) between caudate and hippocampus (area chosen, for display purposes, among all the structures activated in this analysis, see Table 2–3) is correlated with subsequent gains in performance in the SG. This regression analysis indicates that increased strength of the competitive interaction between the caudate nucleus and this cerebral network, including hippocampo-cortical areas, when performance is variable, results in increased overnight gain in performance. After sleep deprivation, this relationship fails to predict subsequent performance gains.

**Table 3:** Functional results for the main effect of session on the trained sequence (Retest – Training).

Area	x mm	y mm	z mm	Z	p_svc_
**SG**					
Left Superior Frontal Cortex	−16	46	50	3.65	**0.014**
Left Anterior Hippocampus	−18	−14	−28	3.53	**0.019**
Left Medial Prefrontal Cortex	−8	66	8	3.27	**0.039**
Right Medial Temporal Cortex	58	−8	−24	3.18	**0.048**
Left Cerebellar Lobule V/VI	−18	−56	−24	3.19	**0.047**
**SDG**					
Right Ventral Putamen	24	4	−20	3.78	**0.009**
Left Posterior Cingulate Cortex	−8	−42	46	3.27	**0.035**
Right Anterior Cingulate Cortex	8	50	4	3.54	**0.017**
**SG (EM SDG)**					
Left Medial Frontal Cortex	−20	42	52	3.41	**0.039**
Left Anterior Hippocampus	−18	−14	−28	3.53	**0.019**
Left Medial Prefrontal Cortex	−8	66	8	3.27	**0.039**
Right Medial Temporal Cortex	56	−8	−26	3.15	**0.051**
Left Cerebellar Lobule V/VI	−18	−56	−24	3.19	**0.047**
**SDG (EM SG)**					
Right Ventral Putamen	24	4	−20	3.78	**0.009**
Left Posterior Cingulate Cortex	−8	−44	42	3.27	**0.035**
Right Anterior Cingulate Cortex	8	50	4	3.54	**0.017**

Significant brain responses after correction over small volume of interest (svc) are reported here. EM: Exclusive Mask; SG: Sleep Group; SDG: Sleep Deprived Group.

We performed the same regression analyses with gain in performance computed with the first 5 sequences, which are deemed unaffected by fatigue. Remarkably, the significant regression between the strength of the negative connectivity (competitive interaction) between caudate nucleus and hippocampo-cortical areas, modulated by performance variability, and gains in performance remains significantly better in sleepers relative to sleep deprived subjects even when controlling for possible fatigue effects (Table 2–4). In other words, even if fatigue effects are accounted for, at the individual level, the participants who presented the most important competitive interaction between caudate and hippocampo-cortical areas to control for performance variability had the largest gains in performance speed after sleep.

#### Between-session changes in cerebral response on the learned sequence

**Figure 4:**
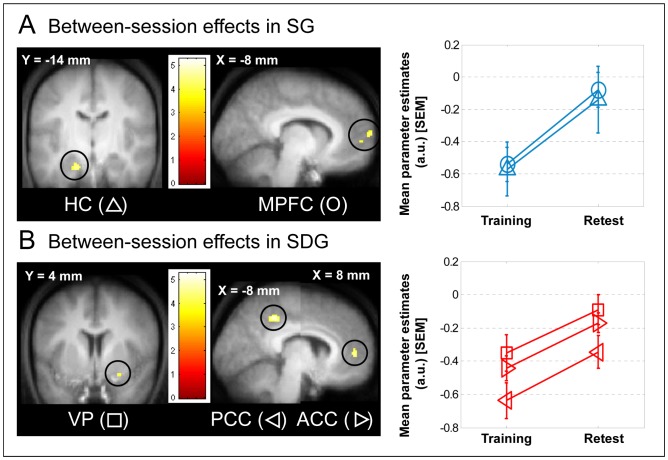
Functional imaging results of the main effect of session on the learned sequence according to the sleep condition (Retest - Training). Functional results are displayed at p_uncorrected_<0.001 over the mean structural image of all subjects. Mean parameter estimates on the trained sequence during training and retest sessions (arbitrary units: a.u.) are presented in the insets where bars represent SEM. HC: Hippocampus, MPFC: Medial Prefrontal Cortex, VP: Ventral Putamen, PCC: Posterior Cingulate Cortex, ACC: Anterior Cingulate Cortex. A- Between-session effects in SG: In sleepers, responses increased in the HC and the MPFC at retest as compared to training. B- Between-session effects in SDG: In sleep-deprived subjects, responses increased from training to retest in the VP and in ACC and PCC.

In the SG, brain responses increased at retest, relative to training, in the left anterior hippocampus, but also in the right posterior hippocampus (but with a more permissive threshold, 38 −24 −20 mm, Z = 2.33, p_svc_ = 0.078) and in polar medial prefrontal cortex (Figure 4A, Table 3). These responses were not observed in the SDG (exclusive mask, Table 3). In contrast, in sleep-deprived subjects, responses increased at retest, relative to training, in the ventral putamen and both anterior and posterior cingulate cortices (Figure 4B, Table 3). These effects were not observed in sleepers (exclusive mask, Table 3).

## Discussion

In this study, we aimed at characterizing the relation between performance variability during initial training and subsequent sleep-dependent motor sequence memory consolidation. We previously showed that activity in the caudate nucleus is correlated with the implementation of performance consistency during initial motor sequence learning [2]. Importantly, the setting of an effective performance mode appears to be driven by a tight interaction between caudate nucleus and frontal areas [2]. Here, using functional connectivity analyses and regression with subsequent changes in performance, we show that the strength of the competitive interaction between the caudate nucleus and a hippocampo-cortical network during initial training can predict subsequent delayed gains in performance after sleep but not after sleep deprivation. This relationship holds irrespective of whether fatigue effects during training are considered in the computation of the overnight changes in performance. After sleep deprivation, the strength of the functional connectivity between these areas no longer predicts later gains in performance, suggesting that these responses are functionally related to memory processing occurring during sleep. We propose that the dynamic large-scale interactions between the striatum and hippocampo-cortical networks, ensuring the reproducibility of sequential motor output during training, may predict subsequent, and possibly sleep-dependent, motor sequence memory consolidation. Finally, changes in responses between training and testing, taken as an indication of offline memory processing, are detected in similar hippocampo-cortical areas after sleep, but not after sleep deprivation.

### Behavior

Our results confirm the effects of sleep and lack of sleep on the consolidation of a recently learned motor sequence [10], [12], [14], [27]. Performance on the trained motor sequence significantly improved at retest when sleep was allowed, but not if it was hindered, on the first post-training night. These results suggest the existence of a particular time-window [28]–[31], here ranging from about 5 to 15 hours after initial training, within which sleep should occur to favor gains in performance. In contrast, sleep taking place, on average, 30 hours after the end of the initial training session (during the second and third post-training nights) does not enhance motor performance. However, one should note the absence of a significant difference in performance gains between groups that may be accounted for by a small, non-significant but continued improvement related to the two recovery nights in the SDG [27], which effectively reduced the sensitivity of the statistics. Furthermore, one limitation of our study in inferring that gains in performance are sleep-dependent is the lack of polysomnographic recordings in the SG.

Our results also confirm that delayed changes in performance are less robust when possible fatigue effects are controlled [17], [18]. However, in our case, the effects of fatigue did not consist in a worsening of performance at the end of training, as reported by Rickard, Brawn and colleagues. Our results still cannot rule out the fact that repetitive practice of the motor task did not induce any fatigue effects at the end of training, which seems unlikely. They rather suggest that fatigue build up during practice may offset the learning effect at the end of training. Importantly, gains in performance remained significant in the SG after controlling for fatigue, indicating that this specific effect was due to an active mnemonic process rather than to a passive dissipation of fatigue [17], [18]. Finally, during the first retest blocks, the absence of fatigue allowed within block improvements in performance that did not differ between groups (i.e., similar to the early training). However, and importantly, average performance speed still significantly improved from training to testing only if sleep was allowed on the first post-training night.

The dynamics of performance variability in this task was detailed in our previous paper [2] and does not progress monotonically during initial training as observed by Adi-Japha and colleagues [1]. The evolution of performance consistency has been described to reflect the implementation of preferential performance modes [1]. Interestingly, performance reproducibility was maintained from training to retest in both groups. These results suggest that the performance mode reached during training represented the sequence of movements in motor memory [1], [2]. The coherent representation specific to the well-mastered sequence created during training was maintained in both groups but triggered gains in performance speed in sleepers but not in sleep deprived subjects.

### Brain Responses Modulated by Decrease in Performance Variability [2]

Before exploring the possible relationship between the implementation of consistent motor behavior during initial training and subsequent motor sequence memory consolidation, we characterized the neural correlates of performance variability during motor sequence acquisition, which had never been done before. For the sake of clarity, these particular results were part of a full and separate publication whose main conclusions are summarized in the following paragraph.

After a fast and substantial increase in consistency during the first part of training, performance suddenly became more variable, followed by a steady decrease in variability during the second part of training [1], [2]. We previously showed that responses in the caudate nucleus were correlated with the particular dynamics of performance consistency such that activity in this area increased as performance became more consistent during initial training. This finding is consistent with the view that the caudate nucleus, involved in associative learning [32], [33], is related to the implementation of preferential performance modes which ensure the reproducibility of sequential motor output during initial training, and is further optimized through practice [34]. Interestingly, the interaction observed between the caudate nucleus and frontal areas was tighter when performance variability was high. Indeed, learning is usually thought to be associated with a progressive shift from the cortical control system to the automatic striatal system, resulting in a systematic and consistent decrease in activity in the controlled network with practice [3]. Accordingly, strong fronto-striatal interactions when performance is highly variable, during early learning, would materialize the influence of sequence representations elaborated in cortical circuits upon striatal representations. A reproducible motor behavior would then be associated with a decrease of cortical weight upon the striatum [2].

### The Strength of the Competitive Interaction between the Caudate Nucleus and Hippocampo-cortical Areas Predicts Subsequent Delayed Gains in Performance after Sleep but not Sleep Deprivation

The strength of the competitive interaction, assessed with functional connectivity analyses, between the caudate nucleus and hippocampo-cortical areas that may participate in the implementation of available performance modes early during learning, is linearly related to subsequent gains in performance observed after sleep but not after sleep deprivation. Importantly, this regression was preserved even after controlling for fatigue effects. Collectively, our results show for the first time, that it is not only activity in hippocampus and striatum [5], but the functional connectivity between these structures that may implement optimal learning and act as a predictor of subsequent, and presumably, sleep-dependent motor sequence memory consolidation.

On one hand, our data indicate that the functional connectivity, which is proportional to performance variability, between caudate nucleus and medial prefrontal cortex (MPFC)/anterior cingulate cortex (ACC) seems to be a predictor of subsequent overnight motor sequence memory consolidation. Recruitment of the MPFC/ACC has already been described in the explicit processing of motor sequential material [26], [35] and in the different processes engaged in sequence generation such as sequence expectation [36], action planning, performance monitoring and error processing, i.e. when there is a need for performance adjustments [37]. More particularly, functional connectivity between the caudate nucleus and the MPFC has been observed in such a way that ACC/MPFC exerts control on the activity of the caudate nucleus during generation of explicitly learned sequences [26]. Early during training, when performance is variable, the MPFC might interact competitively with the caudate nucleus in order to optimize performance monitoring by explicit processes, while the caudate nucleus would progressively implement automatisation under implicit processes [26]. Our results are in line with these findings and further indicate that the strength of the competitive interplay between the MPFC/ACC and the caudate nucleus would participate in the implementation of reproducible motor behavior. Interestingly, our results suggest that these early striato-frontal interactions would also condition offline processes that occur during sleep and induce subsequent gains in performance.

On the other hand, the strength of the competitive interaction between the caudate nucleus and the hippocampus also predicts subsequent overnight motor sequence memory consolidation. We previously showed that activity in the hippocampus during initial oculomotor sequence learning could predict gains in performance occurring overnight but not over the day [5]. We argued that activity in the hippocampus may act as a tag during initial training that would condition subsequent offline processing during sleep [5]. Furthermore, we observed a competitive interaction between the striatum and the hippocampus during initial oculomotor sequence learning [5]. Here we showed that the strength of the competitive interaction between striatum and hippocampus is proportional to performance variability during initial training and conditions the subsequent motor sequence memory consolidation occurring after sleep. The functional significance of the hippocampal responses during initial training has already been discussed in our previous papers [2], [5]. Specifically, these responses may reflect the ability of the hippocampus to associate sequential events during the early phase of training, as already described for motor sequence learning [5], [38]. Furthermore, based on an analogy with spatial memory [39], the recruitment of the hippocampus during early learning would participate in the creation of an allocentric representation of the sequence that is processed during a subsequent sleep period, leading to sleep-dependent enhancement in performance. This hypothesis potentially unifies and explains previous results. First, it would account for the sleep-dependent gains in performance observed if the material to learn requires contextual associations, a process assumed to rely on the hippocampal formation [40]. Second, skill enhancement in an allocentric coordinate frame, i.e. the goal of the sequence, has been known to develop over a period of sleep whereas skill enhancement within an egocentric coordinate frame, i.e. the movements of the sequence, develops independently of sleep [41], [42]. We postulate that the hippocampal-dependent allocentric representation of the sequence might be processed during a subsequent sleep period leading to sleep-dependent enhancement in performance.

In sum, our findings suggest that the dynamical functional interactions between caudate nucleus and hippocampo-cortical areas, ensuring the development of consistent motor behavior during early training, act as a tag for the neuronal populations recruited during learning that contribute to subsequent offline memory processing presumably taking place during sleep of the first post-training night. The nature of the tag is presently unspecified. Some would argue that the increase in synaptic potentiation induced by learning in the hippocampus would require synaptic strength to be downscaled to a baseline level during subsequent (non rapid eye movement) sleep, a mechanism that would eventually consolidate memory [43]. Another hypothesis would assume that hippocampal neuronal ensembles, the connectivity of which was reinforced during training, would participate in memory consolidation by reinforcing synaptic connections with neocortical [44], [45] and striatal [46] structures through experience-dependent replay of neuronal activity during post-training sleep [47]–[50].

### Impact of Sleep and Sleep Deprivation on Offline Cerebral Response Changes

Overnight changes in performance in sleepers where accompanied by increased brain responses at retest, relative to training, not only in the hippocampus, but also in the MPFC. We have already reported the involvement of the hippocampus in overnight retrieval of an oculomotor sequence learning task [5], indicating that the hippocampus not only participates in initial motor sequence learning but also in motor sequence memory retention. Our present data confirm that the hippocampus might be involved in overnight motor sequence memory consolidation. On the other hand, the recruitment of the MPFC has already been described in the explicit processing of motor sequential material [26], [35]. Furthermore, motor sequence consolidation can be accompanied by an overnight enhancement of sequence planning and building in the MPFC [12]. Remarkably, both hippocampus and MPFC showed competitive interaction with the caudate nucleus in proportion to performance variability during initial training; the strength of this interaction predicts subsequent and presumably sleep-dependent motor sequence memory consolidation. These findings suggest that the functional interaction of these areas with the caudate nucleus during initial training forecasts processes that could possibly occur during sleep of the first post-training night and induce an increase in their activity during retest when performance is improved. These results also suggest that these processes specifically occurring during sleep might favor sequence mapping and building at retest through activity in hippocampo-cortical networks. The implication of the MPFC is a novel finding in procedural memory consolidation but this area has been implicated in the early consolidation stages of declarative memories [25], [51], [52]. Our results support the hypothesis that neuronal ensembles, including the hippocampus, tagged during training according to their functional interactions, participate in consolidation of motor sequence memories during subsequent sleep through a reorganization of memories across hippocampal and neocortical areas. This mechanism was previously suspected to underlie consolidation of declarative memories [25], [51], [52] but not procedural memories.

In contrast, for sleep-deprived subjects, in whom motor sequence performance was not enhanced but only stabilized, responses increased at retest, relative to training, in the ventral putamen and both anterior and posterior cingulate cortices. This network is classically described in the long-term retention of motor sequential skills [4]. In line with a study showing increase in task-related putaminal activity after sleep deprivation [14], our results suggest that activity in the striato-cortical network may not depend on sleep. However, our results are not consistent with a recent fMRI study, using a sleep/wake protocol, showing an increase in striatal activity in the sleep group, as compared to the wake group, during the course of motor sequence memory consolidation [15]. It is possible that, in the present study, these striato-cortical networks benefit from the two recovery nights in sleep deprived subjects but our data still suggest that the increase in striato-cortical activity during retest is not dependent on the sleep of the first post-training night. Rather, our data suggest that this striato-cortical network is engaged as a parallel process which stabilizes motor sequence memories over time [8] and prevails when sleep deprivation follows training.

### Conclusions

Motor sequence acquisition implies dynamic large scale interactions between distributed cerebral areas including the striatum, the hippocampus and the prefrontal cortex. Remarkably, these early representations, ensuring the implementation of reproducible motor behavior during initial learning, have a major predictive impact on subsequent, possibly sleep-dependent, motor sequence memory consolidation. Future research should specifically characterize the distinct roles of these two essential structures (hippocampus and striatum) in motor sequence memory consolidation.

## References

[1] Adi-Japha E, Karni A, Parnes A, Loewenschuss I, Vakil E (2008) A shift in task routines during the learning of a motor skill: Group-averaged data may mask critical phases in the individuals’ acquisition of skilled performance. J Exp Psychol Learn Mem Cogn 34: 1544–1551. 18980413 10.1037/a0013217

[2] Albouy G, Sterpenich V, Vandewalle G, Darsaud A, Gais S, et al (2012) Neural correlates of performance variability during motor sequence acquisition. Neuroimage 60: 324–331. 22227134 10.1016/j.neuroimage.2011.12.049

[3] Chein JM, Schneider W (2005) Neuroimaging studies of practice-related change: fMRI and meta-analytic evidence of a domain-general control network for learning. Brain Res Cogn Brain Res 25: 607–623. 16242923 10.1016/j.cogbrainres.2005.08.013

[4] Doyon J, Benali H (2005) Reorganization and plasticity in the adult brain during learning of motor skills. Curr Opin Neurobiol 15: 161–167. 15831397 10.1016/j.conb.2005.03.004

[5] Albouy G, Sterpenich V, Balteau E, Vandewalle G, Desseilles M, et al (2008) Both the hippocampus and striatum are involved in consolidation of motor sequence memory. Neuron 58: 261–272. 18439410 10.1016/j.neuron.2008.02.008

[6] Orban P, Peigneux P, Lungu O, Albouy G, Breton E, et al (2010) The multifaceted nature of the relationship between performance and brain activity in motor sequence learning. Neuroimage 49: 694–702. 19732838 10.1016/j.neuroimage.2009.08.055

[7] McGaugh JL (2000) Memory–a century of consolidation. Science 287: 248–251. 10634773 10.1126/science.287.5451.248

[8] Robertson EM, Pascual-Leone A, Miall RC (2004) Current concepts in procedural consolidation. Nat Rev Neurosci 5: 576–582. 15208699 10.1038/nrn1426

[9] Maquet P (2001) The role of sleep in learning and memory. Science 294: 1048–1052. 11691982 10.1126/science.1062856

[10] Robertson EM, Pascual-Leone A, Press DZ (2004) Awareness modifies the skill-learning benefits of sleep. Curr Biol 14: 208–212. 14761652 10.1016/j.cub.2004.01.027

[11] Press DZ, Casement MD, Pascual-Leone A, Robertson EM (2005) The time course of off-line motor sequence learning. Brain Res Cogn Brain Res. 10.1016/j.cogbrainres.2005.05.010 15990282

[12] Walker MP, Stickgold R, Alsop D, Gaab N, Schlaug G (2005) Sleep-dependent motor memory plasticity in the human brain. Neuroscience 133: 911–917. 15964485 10.1016/j.neuroscience.2005.04.007

[13] Korman M, Raz N, Flash T, Karni A (2003) Multiple shifts in the representation of a motor sequence during the acquisition of skilled performance. Proc Natl Acad Sci U S A 100: 12492–12497. 14530407 10.1073/pnas.2035019100 PMC218785

[14] Fischer S, Nitschke MF, Melchert UH, Erdmann C, Born J (2005) Motor memory consolidation in sleep shapes more effective neuronal representations. J Neurosci 25: 11248–11255. 16339020 10.1523/JNEUROSCI.1743-05.2005 PMC6725908

[15] Debas K, Carrier J, Orban P, Barakat M, Lungu O, et al (2010) Brain plasticity related to the consolidation of motor sequence learning and motor adaptation. Proc Natl Acad Sci U S A 107: 17839–17844. 20876115 10.1073/pnas.1013176107 PMC2955095

[16] Albouy G, Ruby P, Phillips C, Luxen A, Peigneux P, et al (2006) Implicit oculomotor sequence learning in humans: Time course of offline processing. Brain Res 1090: 163–171. 16677617 10.1016/j.brainres.2006.03.076

[17] Rickard TC, Cai DJ, Rieth CA, Jones J, Ard MC (2008) Sleep does not enhance motor sequence learning. J Exp Psychol Learn Mem Cogn 34: 834–842. 18605872 10.1037/0278-7393.34.4.834

[18] Brawn TP, Fenn KM, Nusbaum HC, Margoliash D (2010) Consolidating the effects of waking and sleep on motor-sequence learning. J Neurosci 30: 13977–13982. 20962219 10.1523/JNEUROSCI.3295-10.2010 PMC2978076

[19] Oldfield RC (1971) The assessment and analysis of handedness: the Edinburgh inventory. Neuropsychologia 9: 97–113. 5146491 10.1016/0028-3932(71)90067-4

[20] Buysse DJ, Reynolds CF 3rd, Monk TH, Berman SR, Kupfer DJ (1989) The Pittsburgh Sleep Quality Index: a new instrument for psychiatric practice and research. Psychiatry Res 28: 193–213. 2748771 10.1016/0165-1781(89)90047-4

[21] Gitelman DR, Penny WD, Ashburner J, Friston KJ (2003) Modeling regional and psychophysiologic interactions in fMRI: the importance of hemodynamic deconvolution. Neuroimage 19: 200–207. 12781739 10.1016/s1053-8119(03)00058-2

[22] Lehericy S, Bardinet E, Tremblay L, Van de Moortele PF, Pochon JB, et al (2006) Motor control in basal ganglia circuits using fMRI and brain atlas approaches. Cereb Cortex 16: 149–161. 15858164 10.1093/cercor/bhi089

[23] Penhune VB, Doyon J (2005) Cerebellum and M1 interaction during early learning of timed motor sequences. Neuroimage 26: 801–812. 15955490 10.1016/j.neuroimage.2005.02.041

[24] Penhune VB, Doyon J (2002) Dynamic cortical and subcortical networks in learning and delayed recall of timed motor sequences. J Neurosci 22: 1397–1406. 11850466 10.1523/JNEUROSCI.22-04-01397.2002 PMC6757579

[25] Sterpenich V, Albouy G, Boly M, Vandewalle G, Darsaud A, et al (2007) Sleep-Related Hippocampo-Cortical Interplay during Emotional Memory Recollection. PLoS Biol 5: e282. 17958471 10.1371/journal.pbio.0050282 PMC2039770

[26] Destrebecqz A, Peigneux P, Laureys S, Degueldre C, Del Fiore G, et al (2005) The neural correlates of implicit and explicit sequence learning: Interacting networks revealed by the process dissociation procedure. Learn Mem 12: 480–490. 16166397 10.1101/lm.95605 PMC1240060

[27] Walker MP, Brakefield T, Seidman J, Morgan A, Hobson JA, et al (2003) Sleep and the time course of motor skill learning. Learn Mem 10: 275–284. 12888546 10.1101/lm.58503 PMC202318

[28] Stickgold R, James L, Hobson JA (2000) Visual discrimination learning requires sleep after training. Nat Neurosci 3: 1237–1238. 11100141 10.1038/81756

[29] Maquet P, Schwartz S, Passingham R, Frith C (2003) Sleep-related consolidation of a visuomotor skill: brain mechanisms as assessed by functional magnetic resonance imaging. J Neurosci 23: 1432–1440. 12598632 10.1523/JNEUROSCI.23-04-01432.2003 PMC6742239

[30] Smith C (1995) Sleep states and memory processes. Behav Brain Res 69: 137–145. 7546305 10.1016/0166-4328(95)00024-n

[31] Hennevin E, Hars B, Maho C, Bloch V (1995) Processing of learned information in paradoxical sleep: relevance for memory. Behav Brain Res 69: 125–135. 7546303 10.1016/0166-4328(95)00013-j

[32] Moses SN, Brown TM, Ryan JD, McIntosh AR (2010) Neural system interactions underlying human transitive inference. Hippocampus 20: 894–901. 20054816 10.1002/hipo.20735

[33] Lehericy S, Benali H, Van de Moortele PF, Pelegrini-Issac M, Waechter T, et al (2005) Distinct basal ganglia territories are engaged in early and advanced motor sequence learning. Proc Natl Acad Sci U S A 102: 12566–12571. 16107540 10.1073/pnas.0502762102 PMC1194910

[34] Rickard TC (1999) A CMPL alternative account of practice effects in numerosity judgement tasks. Journal of Experimental Psychology: Learning, Memory, and Cognition 25: 532–542.

[35] Destrebecqz A, Peigneux P, Laureys S, Degueldre C, Del Fiore G, et al (2003) Cerebral correlates of explicit sequence learning. Brain Res Cogn Brain Res 16: 391–398. 12706219 10.1016/s0926-6410(03)00053-3

[36] Koechlin E, Corrado G, Pietrini P, Grafman J (2000) Dissociating the role of the medial and lateral anterior prefrontal cortex in human planning. Proc Natl Acad Sci U S A 97: 7651–7656. 10852964 10.1073/pnas.130177397 PMC16600

[37] Ridderinkhof KR, van den Wildenberg WP, Segalowitz SJ, Carter CS (2004) Neurocognitive mechanisms of cognitive control: the role of prefrontal cortex in action selection, response inhibition, performance monitoring, and reward-based learning. Brain Cogn 56: 129–140. 15518930 10.1016/j.bandc.2004.09.016

[38] Schendan HE, Searl MM, Melrose RJ, Stern CE (2003) An FMRI study of the role of the medial temporal lobe in implicit and explicit sequence learning. Neuron 37: 1013–1025. 12670429 10.1016/s0896-6273(03)00123-5

[39] Doeller CF, King JA, Burgess N (2008) Parallel striatal and hippocampal systems for landmarks and boundaries in spatial memory. Proc Natl Acad Sci U S A 105: 5915–5920. 18408152 10.1073/pnas.0801489105 PMC2311337

[40] Spencer RM, Sunm M, Ivry RB (2006) Sleep-dependent consolidation of contextual learning. Curr Biol 16: 1001–1005. 16713957 10.1016/j.cub.2006.03.094

[41] Cohen DA, Pascual-Leone A, Press DZ, Robertson EM (2005) Off-line learning of motor skill memory: a double dissociation of goal and movement. Proc Natl Acad Sci U S A 102: 18237–18241. 16330773 10.1073/pnas.0506072102 PMC1312380

[42] Albouy G, Fogel S, Pottiez H, Nguyen VA, Ray L, et al (2013) Daytime sleep enhances consolidation of the spatial but not motoric representation of motor sequence memory. PLoS One 8: e52805. 23300993 10.1371/journal.pone.0052805 PMC3534707

[43] Tononi G, Cirelli C (2003) Sleep and synaptic homeostasis: a hypothesis. Brain Res Bull 62: 143–150. 14638388 10.1016/j.brainresbull.2003.09.004

[44] Ji D, Wilson MA (2007) Coordinated memory replay in the visual cortex and hippocampus during sleep. Nat Neurosci 10: 100–107. 17173043 10.1038/nn1825

[45] Euston DR, Tatsuno M, McNaughton BL (2007) Fast-forward playback of recent memory sequences in prefrontal cortex during sleep. Science 318: 1147–1150. 18006749 10.1126/science.1148979

[46] Lansink CS, Goltstein PM, Lankelma JV, McNaughton BL, Pennartz CM (2009) Hippocampus leads ventral striatum in replay of place-reward information. PLoS Biol 7: e1000173. 19688032 10.1371/journal.pbio.1000173 PMC2717326

[47] Rasch B, Buchel C, Gais S, Born J (2007) Odor cues during slow-wave sleep prompt declarative memory consolidation. Science 315: 1426–1429. 17347444 10.1126/science.1138581

[48] Peigneux P, Laureys S, Fuchs S, Collette F, Perrin F, et al (2004) Are spatial memories strengthened in the human hippocampus during slow wave sleep? Neuron 44: 535–545. 15504332 10.1016/j.neuron.2004.10.007

[49] Maquet P, Laureys S, Peigneux P, Fuchs S, Petiau C, et al (2000) Experience-dependent changes in cerebral activation during human REM sleep. Nat Neurosci 3: 831–836. 10903578 10.1038/77744

[50] Rudoy JD, Voss JL, Westerberg CE, Paller KA (2009) Strengthening individual memories by reactivating them during sleep. Science 326: 1079. 19965421 10.1126/science.1179013 PMC2990343

[51] Gais S, Albouy G, Boly M, Dang-Vu TT, Darsaud A, et al (2007) Sleep transforms the cerebral trace of declarative memories. Proc Natl Acad Sci U S A 104: 18778–18783. 18000060 10.1073/pnas.0705454104 PMC2141853

[52] Takashima A, Petersson KM, Rutters F, Tendolkar I, Jensen O, et al (2006) Declarative memory consolidation in humans: a prospective functional magnetic resonance imaging study. Proc Natl Acad Sci U S A 103: 756–761. 16407110 10.1073/pnas.0507774103 PMC1334654

